# Towards human-computer synergetic analysis of large-scale biological data

**DOI:** 10.1186/1471-2105-14-S14-S10

**Published:** 2013-10-09

**Authors:** Rahul Singh, Hui Yang, Ben Dalziel, Daniel Asarnow, William Murad, David Foote, Matthew Gormley, Jonathan Stillman, Susan Fisher

**Affiliations:** 1 Department of Computer Science, San Francisco State University, San Francisco, CA, USA; 2 Center for Discovery and Innovation in Parasitic Diseases, University of California, San Francisco, CA, USA; 3 Open University Program, San Francisco State University, CA, USA; 4 Department of Obstetrics, Gynecology, and Reproductive Sciences, University of California, San Francisco, CA, USA; 5 Romberg Tiburon Center and Department of Biology, San Francisco State University, San Francisco, CA, USA; 6 Department of Integrative Biology, University of California Berkeley, CA, USA; 7 Department of Anatomy and Pharmaceutical Chemistry, University of California, San Francisco, San Francisco, CA, USA

## Abstract

**Background:**

Advances in technology have led to the generation of massive amounts of complex and multifarious biological data in areas ranging from genomics to structural biology. The volume and complexity of such data leads to significant challenges in terms of its analysis, especially when one seeks to generate hypotheses or explore the underlying biological processes. At the state-of-the-art, the application of automated algorithms followed by perusal and analysis of the results by an expert continues to be the predominant paradigm for analyzing biological data. This paradigm works well in many problem domains. However, it also is limiting, since domain experts are forced to apply their instincts and expertise such as contextual reasoning, hypothesis formulation, and exploratory analysis *after *the algorithm has produced its results. In many areas where the organization and interaction of the biological processes is poorly understood and exploratory analysis is crucial, what is needed is to integrate domain expertise *during *the data analysis process and use it to drive the analysis itself.

**Results:**

In context of the aforementioned background, the results presented in this paper describe advancements along two methodological directions. *First*, given the context of biological data, we utilize and extend a design approach called experiential computing from multimedia information system design. This paradigm combines information visualization and human-computer interaction with algorithms for exploratory analysis of large-scale and complex data. In the proposed approach, emphasis is laid on: (1) allowing users to directly visualize, interact, experience, and explore the data through interoperable visualization-based and algorithmic components, (2) supporting unified query and presentation spaces to facilitate experimentation and exploration, (3) providing external contextual information by assimilating relevant supplementary data, and (4) encouraging user-directed information visualization, data exploration, and hypotheses formulation. *Second*, to illustrate the proposed design paradigm and measure its efficacy, we describe two prototype web applications. The first, called XMAS (Experiential Microarray Analysis System) is designed for analysis of time-series transcriptional data. The second system, called PSPACE (Protein Space Explorer) is designed for holistic analysis of structural and structure-function relationships using interactive low-dimensional maps of the protein structure space. Both these systems promote and facilitate human-computer synergy, where cognitive elements such as domain knowledge, contextual reasoning, and purpose-driven exploration, are integrated with a host of powerful algorithmic operations that support large-scale data analysis, multifaceted data visualization, and multi-source information integration.

**Conclusions:**

The proposed design philosophy, combines visualization, algorithmic components and cognitive expertise into a seamless processing-analysis-exploration framework that facilitates sense-making, exploration, and discovery. Using XMAS, we present case studies that analyze transcriptional data from two highly complex domains: gene expression in the placenta during human pregnancy and reaction of marine organisms to heat stress. With PSPACE, we demonstrate how complex structure-function relationships can be explored. These results demonstrate the novelty, advantages, and distinctions of the proposed paradigm. Furthermore, the results also highlight how domain insights can be combined with algorithms to discover meaningful knowledge and formulate evidence-based hypotheses during the data analysis process. Finally, user studies against comparable systems indicate that both XMAS and PSPACE deliver results with better interpretability while placing lower cognitive loads on the users. XMAS is available at: http://tintin.sfsu.edu:8080/xmas. PSPACE is available at: http://pspace.info/.

## Background

Advances in high-throughput techniques have led to an exponential growth in the amount of information being generated in life sciences. To understand and model the underlying biology, scientists therefore have to often seek out patterns in a sea of data. For such tasks, traditionally, one of two types of approaches has been used: the first involves statistical and algorithmic methods and the second, visualization-based data analysis. The philosophies underlying the aforementioned classes of techniques are often conflicting; algorithmic methods are characterized by attributes of automation and large-scale analysis. The exclusive use of algorithms works when the patterns being sought for are well understood and can be identified through precise step-by-step processing. On the other hand, visualization-based methods take advantage of cognitive strengths in pattern recognition and help in exploratory analysis, hypotheses formulation, and sense-making. In spite of their differences, it may be valuable to take strengths of both approaches for creating novel discovery paradigms and tools [1].

In this paper we describe the adaptation to biological data analysis of a design approach called experiential computing. Originating in the area of multimedia information system design [2-6], experiential computing supports data analysis by combining user expertise with information visualization and algorithms in a tight loop. We also study two biological data analysis systems based on this design paradigm. The first of these, called XMAS, is directed towards analysis of time-series expression data. The second system, called PSPACE, is directed towards holistic structure-function analysis of large groups of structures through creating interactive maps of the protein structure space. Each of these systems illustrate how design principles proposed by us can be translated for different types of biological data. In the remaining part of this section, we review the prior research directions in the above two biological areas and identify the distinctions and contributions of our research.

### Microarray information visualization and analysis

We begin with the issue of exploratory analysis of microarray data. In this context, a host of algorithmic frameworks such as Bayesian belief networks, various forms of unsupervised clustering, and machine learning techniques have been proposed [7-9] and a number of review papers that characterize the entire area are now available (see for instance [8,10]). Many of these methods are significantly sophisticated both in terms of their mathematical modeling as well as the underlying algorithms. However, they function primarily as a "black box" giving domain experts very limited control over the analysis process. Consequently, within these methods, little support is made available for user-guided exploration and hypotheses formulation. Given that the end users are often domain specialists with years of know-how, a purely algorithmic process may end up underutilizing the available human knowledge, experience and contextual reasoning ability - all of which can help explore alternatives unforeseen by the algorithm designers.

A number of tools have been either explicitly designed or can be adapted for visualization of microarray data. In Clusterview [11], a heat-map was used to display the gene expression pattern. Parallel-coordinate visualization was employed in Timesearcher [12] and dendograms were used as the primary visualization mode in HCE [13] to present results from hierarchical clustering (other data visualization modalities such as heat maps, parallel coordinate displays, scatterplots, and histograms were also supported). Since clustering is a common data mining technique, several tools have been developed specifically for visualizing clustering results, such as TreeMap [14], Genesis [15], and TM4 [16]. In Time-series Explorer [17], coordinated visualizations (e.g. scatter-plots) reflecting changes in gene activity were used to explore the data. In [14], the TreeMap paradigm was extended to visualize and query microarray data using the Gene Ontology (GO) framework. Numerous plug-ins have also been developed for existing software systems such as the BioConductor package for R [18], along with SAM [19] and PAM [20] for Excel. Finally, software packages such as ArrayTrack [21], Spotfire [22] and GeneSpring [23] offer various computational and visualization tools for microarray data analysis. Compared with algorithm-oriented methods, visualization-based systems provide intuitive representations of the results produced by a specific algorithm and allow users to interact with the visualized results. However, these methods primarily focus on visualizing the final results after an algorithm has been applied and rarely support analysis by integrating the algorithms and user expertise in a closed loop. Efforts that have attempted to integrate algorithms with visualization include [24], where *k*-means gene-based clustering and autoregression analysis were employed to model regulatory interactions. Visualization was then employed for finding unknown gene interactions. The idea of combining statistical and visualization methods was also utilized in the GeneVAnD system [25].

### Visualization and analysis of maps of the protein structure space

Mirroring the growth of transcriptional information, the protein structure initiative and other related efforts, have led to the availability of a large number of solved structures. The presence of such, hitherto unavailable amount of structural information can allow us to reason about the very topology of structure space. Furthermore, contextual and comparative analysis of proteins and their structural neighbors may yield insight into structure-function relationships unavailable to isolated investigations or pairwise comparisons of protein structures alone.

The theoretical foundations and algorithms for mapping and analysis of collections of proteins to a low-dimensional space involve either low dimensional projections of all-by-all distance matrices of structures [26-28] or dimensionality reduction of vector-space representation of proteins [29]. However, unlike the case of transcriptional data, there are no dedicated software systems for visualization and analysis of maps of protein structure space (hereafter abbreviated as MPSS).

### Experiential computing: integrating visualization, algorithms, and the user

Supporting information processing, once it moves out of the realm of precisely specifiable queries and enters the exploratory domain requires facilitating the integration of user-centric capabilities and powerful algorithms. Towards this goal, in multimedia computing, the principles of experiential computing were proposed to address the challenges of information exploration, assimilation, and retrieval in settings involving heterogeneous and multifarious data [2-6]. Experiential computing argues for the design of systems where users can apply their natural senses to observe, interact with, and explore the data. We characterize experiential systems by the following properties: (1) they are direct, in that they do not use complex metaphors or commands either for presentation of the information or for mediating interactions with it, (2) they support the same query and presentation spaces so as to provide intuitive and direct user-data interactions, (3) they maintain user state and context, (4) they present information independent of (but not excluding) different data sources, (5) they provide multiple semantic perspectives on the data, both for presentation and interactions, and (6) they seamlessly integrate powerful algorithmic analysis with visualization and interaction.

Experiential computing shares many characteristics with ideas proposed in visualization research, and consequently, should be thought of as a paradigm that encapsulates information visualization. The uniqueness of this paradigm lies in the emphasis it places on facilitating human-machine synergy by combining powerful algorithmic data processing and analysis with interfaces that allow users to leverage their perceptual abilities for exploration and assimilation. This design principle is supported by the cognitive fit theory [30], which suggests that users achieve better task performance when they do not need to transform the model through which information is presented to a different mental model, in order to solve a task. Furthermore, Perer and Shneiderman [31], have recently argued the importance of incorporating (statistical) computing in visualization for exploratory data analysis. While the focus of this paper is on analysis of biological data, we have also applied the experiential computing paradigm to design information systems for storage and querying of data from high-throughput drug screening [32-34].

In the following we describe the design principles of experiential computing using two systems: XMAS (eXperiential Microarray Analysis System) and PSPACE (Protein Structure Space Explorer). Each of these systems represents advancements to the state of the art: XMAS supports complex temporal pattern analysis data through a combination of visualization and algorithms while PSPACE is currently the only available system for visualization and user-driven analysis of protein structure space maps. While these systems are crucial to our narrative, our central goal is to use them as examples that underline the principles of experiential computing as a design paradigm for analysis of complex biological information.

## Methods and software implementations

### Design principles and key design features of XMAS

The primary goal of XMAS is to promote exploration, hypotheses formulation, and knowledge discovery by integrating the user directly in an interactive and reflexive visualization environment with powerful algorithmic capabilities. An early version of XMAS was reported in [35]. The latest version of XMAS has the following key functional features:

1. A graphical user interface (GUI) that supports direct point-and-click interactions with the data. The GUI avoids the use of complex metaphors and commands for visualization and interactions with the data. The GUI also minimizes the cognitive load on the user by presenting user's input queries with their corresponding results in a unified query-visualization-interaction space.

2. Persistence of user and data context since abrupt context switching is known to degrade user performance and experience.

3. A reflexive interaction environment, where data can be observed from multiple, semantically correlated views. Furthermore, changes in any view brought through purposive user manipulations are propagated, in real time, to the other views. This allows users to intuitively perceive the underlying relationships between the different semantic views supported by the data.

4. Integration of external contextual information through assimilation of a variety of supplementary data sources such as pathway data from the Kyoto Encyclopaedia of Genes and Genomes (KEGG).

5. An integrated set of algorithmic operators including operators for data clustering, trajectory-based analysis, cross-data comparisons, and numerical and statistical data analysis. These operators can be invoked by users on-demand, based on their individual exploratory goals and contexts, without adhering to a predefined processing workflow.

Most operators in XMAS are interoperable and can be applied in a user-directed sequence. Thus, users can interactively respond to the analysis outcomes and design their own explorative paths to reason about the data. Next, we describe the key components and the implementation strategies of XMAS as follows: (1) the input data of XMAS, (2) an overview of the XMAS user interface, (3) operators for data preprocessing and integration, and (4) operators for data analysis.

### Input data for XMAS

XMAS accepts logically heterogeneous data types, including time series microarray data, supplementary data, and user-defined annotations. A time series microarray dataset is often composed of thousands of short time series, each of which portrays the temporal trend of expression level exhibited by a gene (or probe) during the study. To increase the experiment reliability and reduce the outlier effect, multiple samples are often employed at each time point. A variety of supplementary data is also accepted by XMAS. Examples of such data include KEGG pathways [36] and probe annotations. Additionally, XMAS also accepts user-defined annotations to enrich data analysis. For example, a set of genes can be annotated for further investigation.

### The XMAS user interface

One of the key requirements of experiential computing is a unified query and presentation space. The XMAS interface pairs versatile and complementary data views and visualizations with operators for analyzing and exploring the data. In the following description, the reader will observe that the key characteristics of the XMAS user interface closely reflect the design principles of experiential computing in that the interface is intuitive, reflexive, and contains a unified query-presentation workspace. This minimizes context switching, which is known to reduce the cognitive load on users [2].

**Figure 1:**
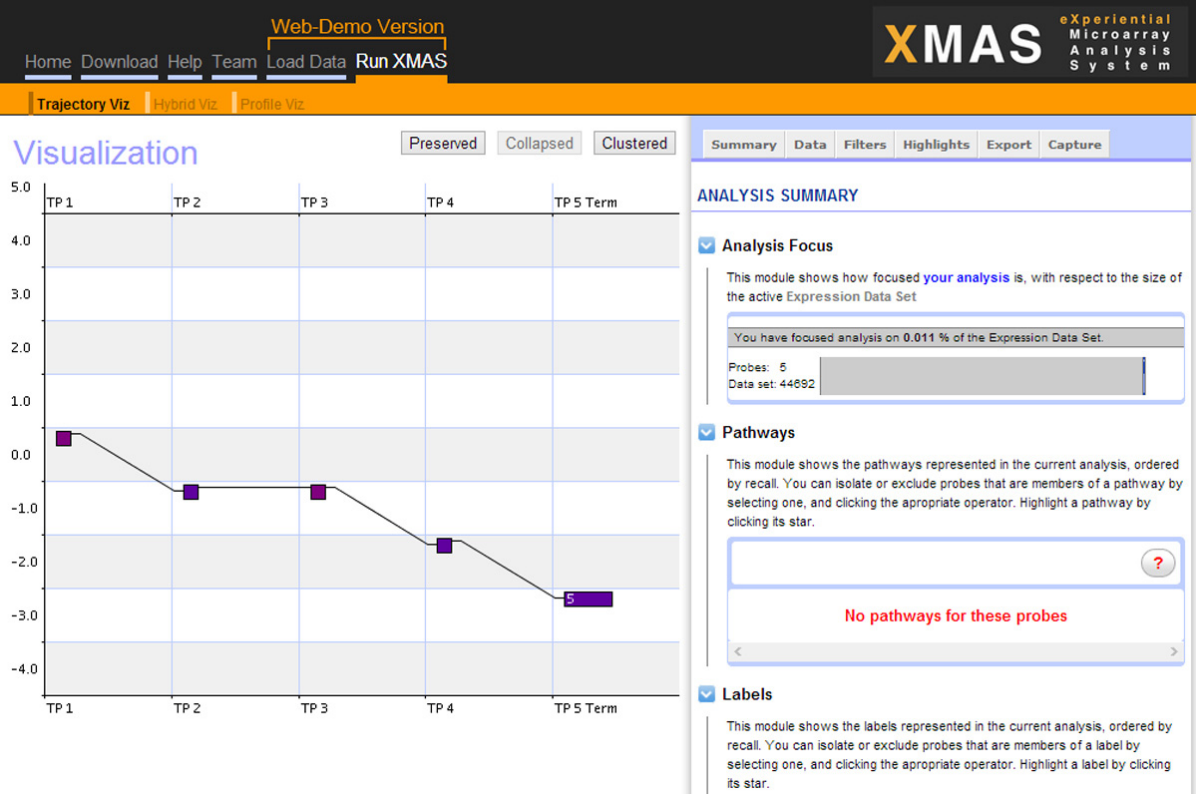
**The XMAS user interface: a unified query and presentation space**. Navigation options are listed horizontally at the top. The primary visualization zone lies on the left and the sidebar zone on the right.

Figure 1 shows the XMAS user interface, which remains consistent throughout data analysis. The site-level navigation links are listed horizontally at the top. Beneath these links are two main visualization zones: the primary zone on the left and the sidebar zone on the right. The use of XMAS only requires clicking on the interface to initiate different data operators or visualizations. Users are granted full control in constructing explorative paths that best suit their specific information or sense-making needs. XMAS also maintains the state and context in terms of both users and data context during analysis. Such contextual information can be saved and resumed (re-loaded) at the user's command. Finally, users can annotate genes considered to be meaningful any time and use these annotations later.

**Figure 2:**
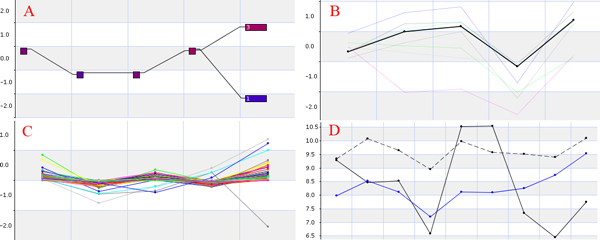
**Visualization types in the primary presentation zone of the XMAS interface**. (A) The trajectory visualization that groups probes with similar discretized expression profiles, (B) The hybrid visualization that shows the expression profiles with the same discretized trajectory in the same color; their mean expression profile is highlighted in bold; (C) The profile visualization that displays up to 1000 un-discretized expression profiles, and (D) The comparative view of selected probes in two comparable datasets.

**Figure 3:**
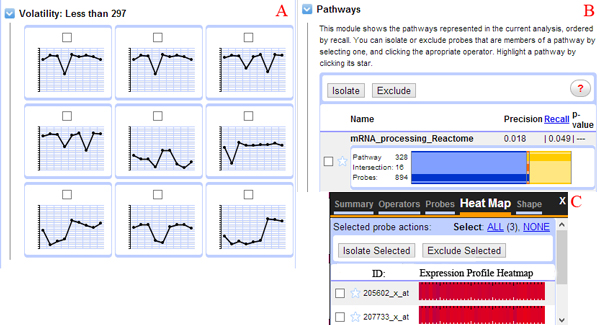
**Main data views in the sidebar zone of the XMAS interface**. (A) Probe expression profiles sorted by volatility, (B) pathway intersection graph, where blue represents the number of probes in the current context, yellow the number of probes in a given pathway and orange the number of common probes, and (C) expression profile heat maps.

Of the two main visualization zones in XMAS, the primary zone is used to display different views of the data. These views are integrated with interoperable data analysis operators. XMAS offers four main types of views as shown in Figure 2. A user can switch among the first three views using the three visualization operators directly below the site-wide navigation links (Figure 1). Detailed information of the underlying data in the primary zone can be obtained by a single click over the visualized data of interest and will be shown through an in-place window. Note that the comparative view becomes valid when both a primary and a secondary dataset are specified. This view shown in Figure 2(D) also includes facilities of subtractive visualization of two data sets, which displays the difference in the expression profile for the same probe in both datasets. Additionally, a "Secondary Dataset Visualization" option is also available, allowing users to analyze separately the secondary dataset loaded in XMAS. This allows a user to analyze both datasets separately thus allowing for a less crowded presentation space. The second important visualization zone, called the sidebar zone contains a hover/drop navigation bar, providing access to diverse data views and operators. Figure 3 shows three main data views available in the sidebar zone.

### Operators in XMAS

XMAS supports different types of operators that can be invoked by the user to perform a spectrum of tasks starting from data loading and pre-processing to complex data analysis. In the following, we describe each of these classes

#### Operators for data loading, pre-processing and integration

*Data loading operator*: XMAS accepts both time series microarray data (base-2 logarithmically transformed) and supplementary data (e.g. probe annotations) in plain text format. To load such data, a user simply selects the folder and uses the load button to call the data loader.

*Data preprocessing operators*: Prior to visualization and analysis, two parameterized preprocessing operators are applied to the time-series microarray data: temporal aggregation and trajectory discretization. The former operator allows a user to "cut" each time series into segments of specific length. It then represents each segment by the aggregated mean or median expression level over all the involved time points. Trajectory discretization transforms gene expression levels to a set of discrete values using an equi-width discretization with a user specified width value. The result of this operator is a collection of discretized time series or probe trajectories, each of which can be loaded and visualized on-demand. All probes with expression levels falling into a specific bin at a given time point form a trajectory node. Each node is connected to a single parent node (from the previous time period) and one or more child nodes (subsequent time period) as shown in Figure 2. By clicking on a node, users can manipulate the list of associated probes. The discretized trajectory representation provides an overview of the entire dataset and can often initiate serendipitous focal analyses. XMAS also supports operations that switch between discretized trajectories and non-discretized ones.

*Data integration operators*: These operators are fully interoperable and can be categorized into the following four types: (1) *Probe-gene integrators: *relate probes and their expression profiles to gene data. (2) *Probe-pathway integrators: *facilitate an awareness of individual probes involved within multiple pathways, and multiple interacting probes in a given pathway. (3) *Probe-label integrators*: allow users to apply their domain knowledge and define labels for certain probes. (4) *Probe-trajectory integrators*: identify probes contained within a trajectory, and the specific trajectory to which a probe belongs.

#### Operators for data analysis

*Expression profile-based operators*: These operators enable users to discover similarities between probe-based expression profiles by analyzing their discretized trajectories. XMAS includes four such operators:

1. *Trajectory shape-based analysis*: this operator finds similarly shaped trajectories regardless of the initial expression level and clusters them together. Probes of the same trajectory shape are essentially co-expressed at each time point, there corresponding to one co-expression pattern. An example of the application of this operator is described in the case studies section.

2. *k-means clustering*: this operator puts probes of similar non discretized trajectories into the same group using the commonly used *k*-means clustering algorithm [37]. The value of *k *is a user specified parameter and the probe-probe similarity is measured by the Euclidian distance between the two probes' expression profiles.

3. *Trajectory identity-based clustering*: This operator identifies the genes showing identical discretized probe trajectories. Two trajectories are defined to be identical if they have the same expression level at every time point.

4. *Trajectory shape-based filtering*: These operators identify trajectories that satisfy user-defined criteria such as a specific expression characteristic, or involvement in a specific pathway. Specific probes, labels and pathways can also be selected and highlighted, isolated, or excluded as part of the analysis. The interface for specifying the desired trajectory allows the query to be visually specified based on the change of expression at a time point relative to the previous time point. This operator can also be used to identify inverse relationships between trajectories which can help in understanding gene co-regulating patterns. The section on case studies illustrates the use of this operator.

*Cross-dataset comparison operators*: Many microarray experiments often require comparing the behavior of a set of probes under different conditions to identify differentially expressed probes (genes). Typically, one of the conditions represents the control and the other the experiment. XMAS offers two main methods for data comparison: (1) A user designates one dataset as primary and the other secondary. Once a focused probe set is characterized, the user can load the counterpart in the secondary dataset for comparison with the primary one; or (2) a user calls the differentiation operator to compute a differential dataset, in which the expression level at each time point indicates the differences between a given probe in the two datasets. This differentiated dataset can then be analyzed to understand the comparative behaviors. Figure 2(D) illustrates the result of invoking this comparison operator.

*Operators for numerical and statistical analysis: *To assist in establishing the validity of observations, XMAS provides a set of statistical and numeric operators. These operators are also useful in cases where the complexity of the data leads to difficulties in visualization or when the data volume of the data complicates interactive interactions. Specifically, XMAS includes four such operators:

1. *p-value based grouping evaluator: *Given a background distribution, the lower the *p*-value, the more unlikely that observing a set of probes associated with each other (e.g., in the same cluster) is due to chance. To illustrate computation of the *p*-value in XMAS, consider a pathway annotation where *N *is the number of probes under study and *D *is the number of probes in a given pathway. Let *n *of the *N *probes be associated with each other by a data operator and *k *out of these *n *probes be in the given pathway. The *p*-value, denoted by *p *in Eq.(1), of this association of *n *probes is defined as:

(1) $$p=\frac{\left(\begin{array}{c} D\\ k \end{array}\right)\left(\begin{array}{c} N-D\\ n-k \end{array}\right)}{\left(\begin{array}{c} N\\ n \end{array}\right)}$$

2. *Precision and recall calculators: *Given a set of probes (or genes) identified through the application of pathway-based trajectory filtering operators, precision and recall are used to gauge the analytical power of such operators by comparing the above set with the set of probes (genes) known to be involved in a pathway.

3. *Trajectory volatility*: Let *T*= {*e_1_, e_2_, ..., e_N_*} represent a discretized trajectory of *N *expression values ordered by time. The volatility of this trajectory *V*(*T*) is calculated as shown in Eq.(2). The volatility can be an indication of interestingness. For example, differentially expressed probes will generally have high volatility. It can also be used to identify potentially erroneous probes since their corresponding trajectories often exhibit higher volatility.

(2) $$V\left(T\right)=\sum_{i=2\ldots N}\left(\left|e_{i}-e_{i-1}\right|\right)$$

4. *Trajectory linear trend: *Following the notation used above, the linear trend of a trajectory *L*(*T*) is defined by Eq.(3). Like volatility, linear trend can provide insights into the relative interestingness of probes.

(3) $$L\left(T\right)=\sum_{i=2\ldots N}\left(e_{i}-e_{i-1}\right)$$

### PSPACE: the protein structure space explorer

PSPACE is a web based software system for experiential exploration of protein structure-function relationships through low (two or three) dimensional maps of the protein fold space, displayed as interactive scatter plots. PSPACE allows for interactive visual data analysis and user driven exploration of annotations from external data sources (e.g. CATH [38] and SCOP [39]), which may be mapped to attributes such as the color of points in the MPSS. The software provides operators for panning, zooming, rotation, structure selection as well as on-demand access to details, such as molecular structure, individual pairwise alignments and nearest-neighbors analysis. Like in XMAS, the PSPACE interface is reflective; brushing and linking operators interconnect different components of the visualization so that selection of a protein in one area is reflected in other visual components, thus providing users with a consistent view of the data set.

A complete MPSS would contain more than 83,000 structures. Current computational limitations make interactive contextual analysis of the entire universe impractical. Further, many solved structures have such high degrees of sequence similarity that their inclusion in such representations provides little comparative value. Therefore, in PSPACE, the protein structure space is represented through "reference sets" such as PDBSelect25, a low redundancy sub-sampling of the PDB containing high quality structures with less than 25% sequence identity. Such references attempt to contain the extents of the known protein universe and thus to serve as a useful "background" for contextual analysis of particular structures.

### The PSPACE data presentation and usage model

The indeterminate and contextual nature of structure space relationships calls for exploratory data analysis methods that leverage human perception for hypothesis formulation and integrative reasoning. PSPACE seeks to map complex and multifarious information sources to intuitive, visual attributes in MPSS, and then permits users to explore data according to their own (expert) sense of what is interesting or informative. For example, PSPACE interactively maps CATH and SCOP annotations to provide perspective on structure-function properties of a protein. Molecular structure views of individual structures and pairwise alignments are also supported. Users of PSPACE may:

• Map structures of interest within the context of existing reference sets through upload of PDB files (for novel structures) or lists of PDB IDs (for available structures).

• Perform functional inference through analysis and exploration of structural proximity of adjacent structures annotated with protein properties (CATH and SCOP annotations).

• Generate MPSS maps using different structural alignment methods to provide alternate representations of structure space (currently Dali, CE, and FATCAT are supported).

• Analyze the nearest neighbors of any selected structure.

• Determine spatial-structural metrics such as spatial density and average relative distances between structures.

• User-driven analysis of MPSS using interactive visualizations. MPSS allow for broad and localized topological analysis of structure-function patterns using interactively mapped CATH and SCOP annotations to provide various protein property views. Molecular structure views of individual structures and pairwise alignments are also supported.

**Figure 4:**
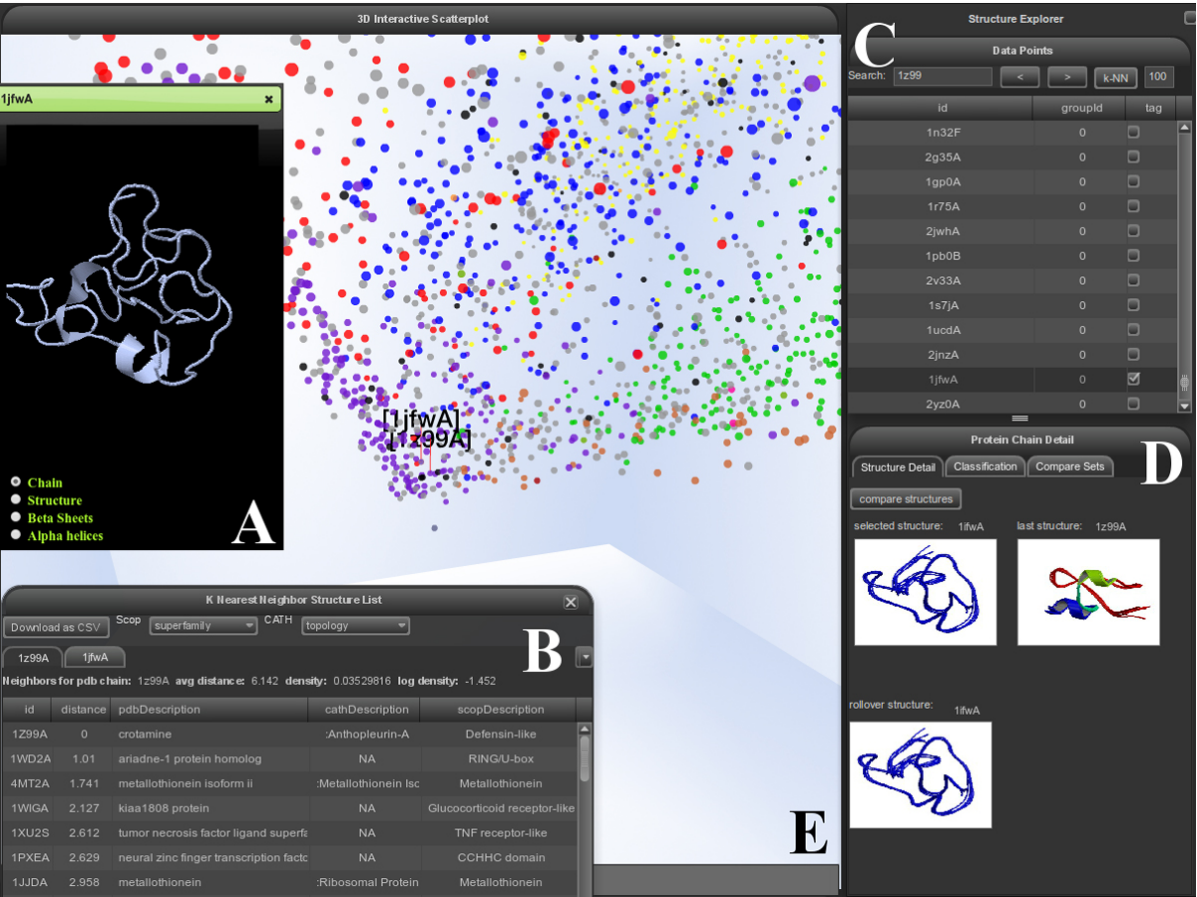
**PSPACE user interface**. Screenshot of PSPACE user interface elements. (A) JMOL structure viewer allowing for 3D structural view of any selected protein chain as well as visualization of aligned comparison between any two protein chains. (B) kNN view showing nearest neighbor list with CATH and SCOP annotations for selected protein chain. (C) Data point view shows currently selected protein chains and allows for search and selection through chain PDB IDs. (D) Protein chain detail view shows JMOL thumbnail images of currently selected protein chains, previously selected structure and structure under the current mouse position. The classification tab of this interface allows for interactive annotation of SCOP and CATH properties at each level of their respective classification hierarchies. The Compare Sets tab provides interface to enable or disable visibility of protein chains for sets of user uploaded structures within the visualization. (E) Three dimensional, interactive scatterplot view showing a FATCAT aligned PDB_SELECT25 reference set of 3,824 protein chains and annotated by SCOP class. This space can be navigated through zoom, rotate, animated navigation to a selected structure, and magnification operators.

**Figure 5:**
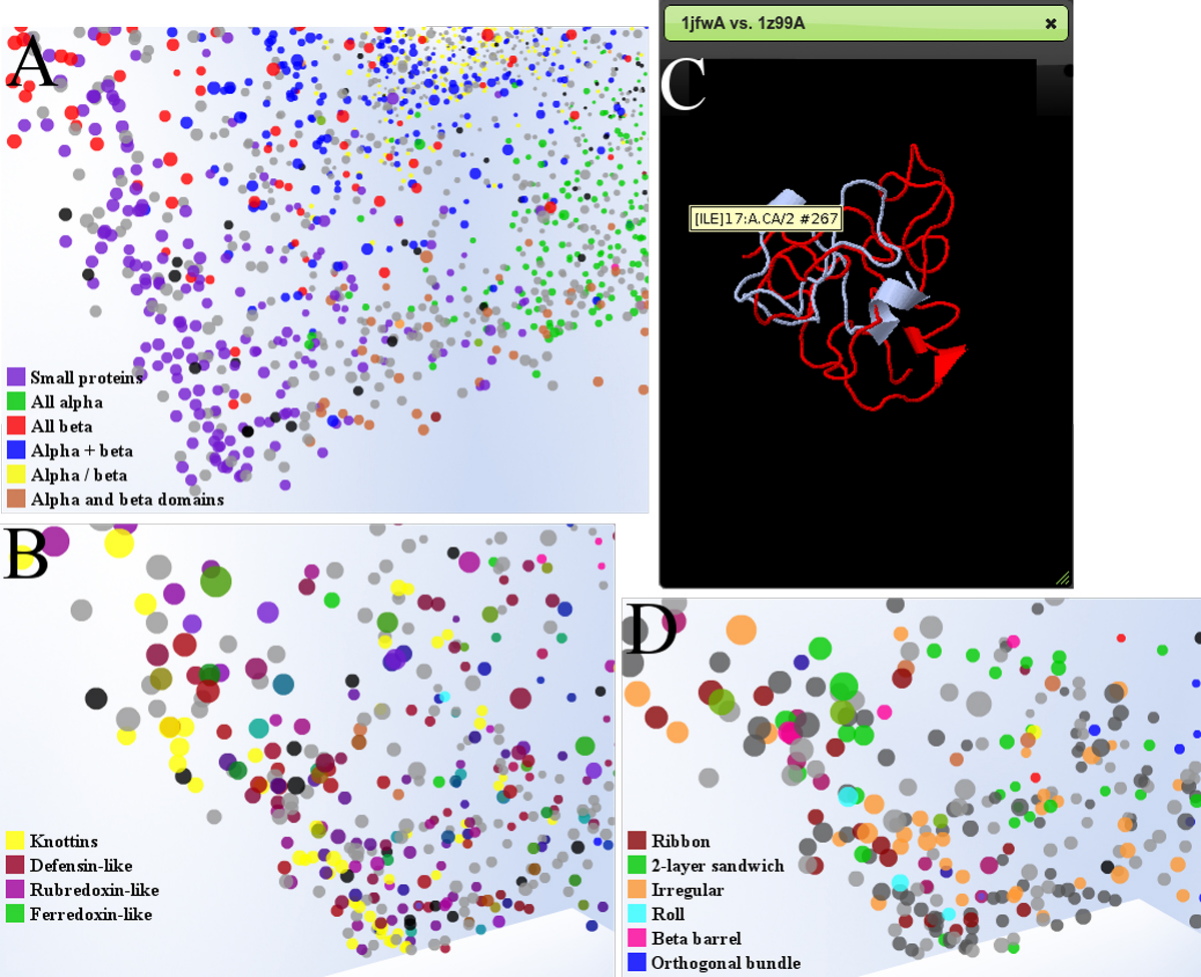
**Sample exploration workflow in PSPACE**. The screenshots presented here show a progressive sequence of steps in a typical workflow using interaction operators in PSPACE. The reference set used in this workflow is the same reference set shown in Figure 5. (A) The FATCAT aligned and SCOP class hierarchy annotated reference set has a tetrahedron shape with mostly small proteins clustered at the apex of the tetrahedron. Zooming in on the apex and annotating the view find clusters of small proteins and peptides. (B) Applying a filter operator to select the SCOP fold hierarchy annotation, and a zoom operator to move closer to structures at the apex, we see that the clusters of small proteins and peptides from the previous view are comprised of knottins, defensin -like and metallothionein chains. (C) Applying a selection operator to two adjacent structures (1JfwA and 1z99A), followed by a JMOL comparison operator generates a JMOL view of the pairwise FATCAT alignment of the two selected structures. (D) Applying the annotation filter operator and selecting CATH architecture shows that most of the structures in this vicinity have not yet been classified by CATH, though for classified structures most appear to be of irregular, ribbon, and roll CATH architectures.

Figure 4 depicts the components of the PSPACE user interface, while Figure 5 shows a sample workflow indicating how PSPACE may be applied to the analysis of structures within the spatial context provided by the MPSS. In Figure 5A, a particular chain is selected in a region corresponding to the small protein class. Interestingly, the small proteins appear to occur at the origin of the MPSS, differentiating into other classes along the axes (see Figure 4E). In Figure 5B, the class annotation is replaced by more specific "fold" level of the SCOP hierarchy, revealing protein fold families occupied by the target protein's structural neighbors. Figure 5C shows the pairwise structure alignment between the target protein and a close neighbor from the metallothionein fold. In Figure 5D, the MPSS has been annotated by CATH architecture. It is also apparent that although most of the visible chains are not found within CATH, Pspace provides a framework for rapidly comparing CATH and SCOP annotations for local MPSS regions which is conducive to "bootstrapping" what information is available into a coherent picture of the protein space surrounding target chains.

## Evaluation and results

In this section we present case studies involving the applications of XMAS on two different expression data sets arising respectively from studies of the human placenta during pregnancy and reaction of marine organisms to heat stress. We also present three different examples of how PSPACE can be used to generate hypotheses about structure-function relationships. Finally, using XMAS and PSPACE as specific realizations of the experiential design paradigm, we present results from user studies that compare the proposed paradigm with other software systems for transcriptional and structural data visualization and analysis. These studies are designed to measure both the complexity and quality of user experiences as well as the efficacy of each of the systems in attaining the goal of the analysis.

### Case study 1: Gene expression analysis at the human maternal interface

The microarray dataset of this study captures the gene expression at the human maternal interface over the course of healthy pregnancy (Geo Accession Number GSE5999) [40]. It includes 45000 expression profiles (probes), representing around 39000 human gene transcripts. Microarray experiments were carried out using 36 non-related placentas (or samples) between the 14^th ^and 40^th ^weeks of pregnancy during 5 intervals: weeks 14-16 (6 samples), 18-19 (9 samples), 21 (6 samples), 23-24 (6 samples), and 37-40 (9 samples). The first four intervals correspond to the mid-gestation stage of pregnancy and the last one to the term stage. The complete experimental protocol used to generate this data is described in [40]. The following supplementary data were integrated into XMAS to enrich the data analysis: detailed probe annotations such as probe-gene correspondence [41,42], Pathways from KEGG and GenMAPP, and a list of differentially expressed genes (DEGs) and human chromosome groupings [40].

**Figure 6:**
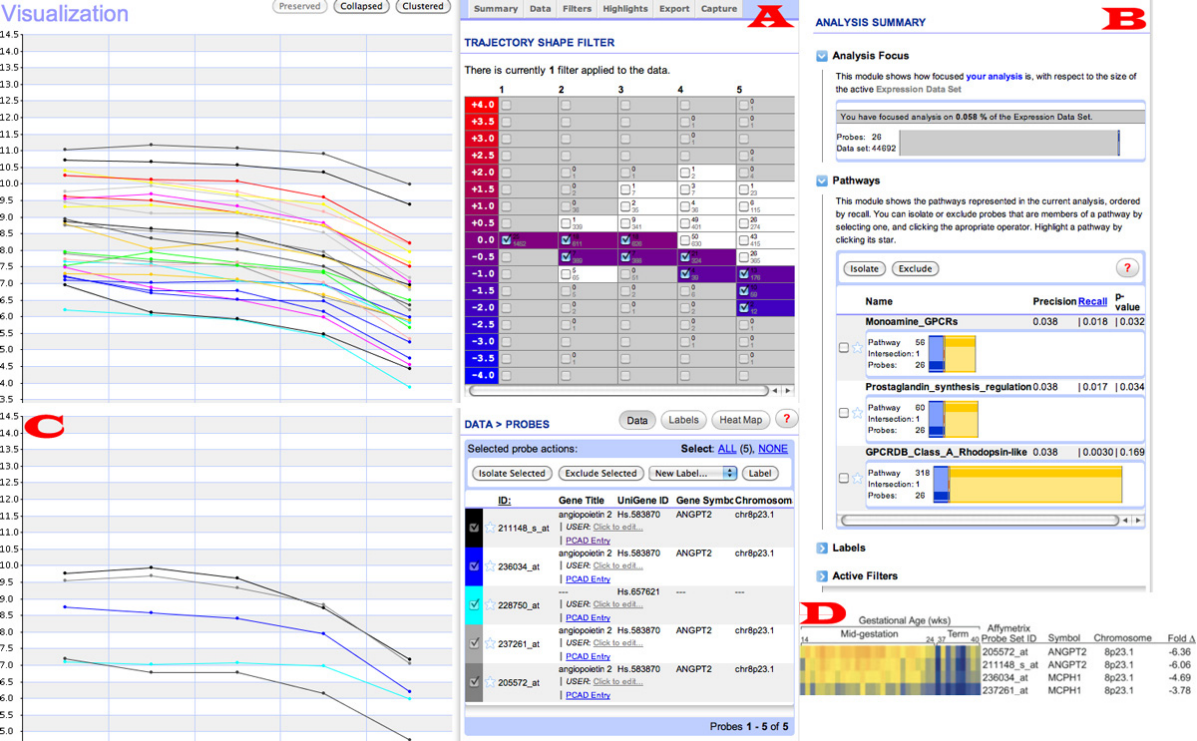
**Case study involving the human maternal interface dataset**. (A) Using the shape-based operator to identify probes showing a significant reduction of expression level at term; (B) the accompanying sidebar view; (C) the highest ranked cluster reported in [27]; and (D) the probes identified at the end of the case study, one of which is previously unknown yet likely a DEG (differentially expressed gene).

We next describe how a biologist analyzed this dataset using XMAS. The initial goal was to identify genes whose expression level significantly reduce at term when the placenta starts to shut down in preparation for delivery. Having loaded the data to XMAS, the biologist selected the median value of the involved samples to represent each period and discretized the trajectories using a bin width of 0.5. To identify genes showing a significant reduction of expression level at term, the trajectory shape-based operator was employed (Figure 6(A)). The sidebar view (Figure 6(B)) presented an integrated visualization of the 26 specific probes of interest. The following observations could be made about these probes: (1) they had a relatively insignificant pathway involvement, (2) of the 26 probes, 15 were labeled as DEGs; and (3) five of the 26 probes originated on the 8^th ^Chromosome, with a *p*-value of 0.005. Based on this contextual information, the biologist decided to look further into the five chromosome-linked probes by clicking on the side bar (Figure 6(C)) and observed that four of these probes were identified as DEGs in literature [40]. The five probes were next compared with the clustering results reported in [40], and it was discovered that the four DEG-labeled probes matched exactly with the highest scoring cluster (Figure 6(D)). The balance of evidence thus indicated that the dashed trajectory could represent a DEG. The biologist therefore determined at this point that the non-DEG probe might actually be differentially expressed and decided to use this as a strong hypothesis and design wet-lab investigations to verify it.

The reader may note that this study demonstrates certain important and desirable features of XMAS: the user (a biologist with no computational background) could integrate his domain expertise and choose a discovery path that best suited his need without complex programming or a steep learning curve. Further, the user could not only verify results from previous studies but also discovered a probe, which corresponded to a likely DEG and was not recognized as such, in an earlier investigation [43].

### Case study 2: Cross-dataset analysis for the heat stress experiment on porcelain crabs

**Figure 7:**
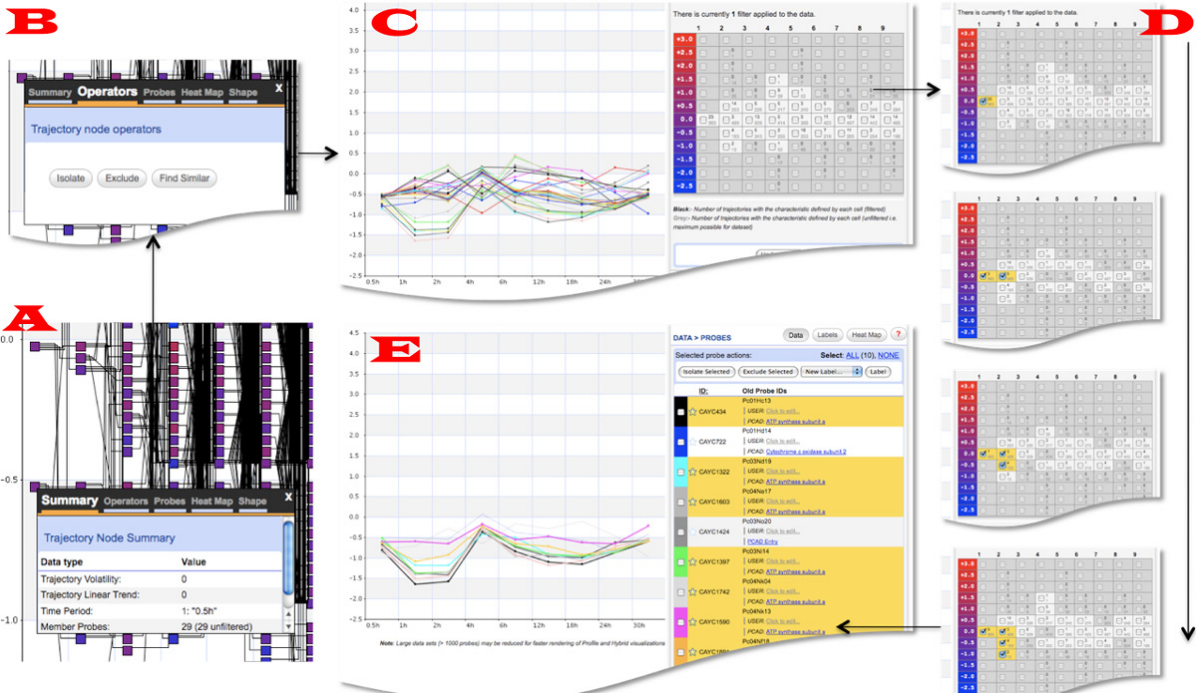
**Revealing probes exhibiting a negative expression differential shortly after heat shock treatment**. Clockwise from bottom left: (A) 29 probes were identified to have a negative expression differential at 0.5 hours, (B) an in-place window was used to isolate the 29 probes, (C) the view of the 29 un-discretized trajectories, (D) the trajectory characteristic based operator was called to refine the probes, and (E) a view of the resulting probes with supplementary data presented in the sidebar.

This study analyzes the genetic response of porcelain crabs reacting to heat stress over a period of 30 hours (GEO Accession Number GSE7498) [44]. In the experiment, crabs were placed in temperature controlled coolers at 11°C (control group) or were thermally ramped from 11°C to 30°C (heat stress group) over a 4 hour (h) period. Following this, crabs were placed in a common recovery aquarium at 11°C and sampled at 9 recovery time points (number of individuals sampled at each time point indicated in brackets): 0.5h (5), 1h (4), 2h (5), 4h (3), 6h (4), 12h (5), 18h (5), 24h (5), and 30h (5). A total of 13,824 probes were included on in-house microarray chips. The complete experimental protocol is presented in [44]. The Porcelain Crab Array Database (PCAD) [45] is directly linked to XMAS to provide an integrated data view during analysis. The biological objective of this study was to expose the genes that were impacted by the heat stress right after the heat stress, i.e., at the first time point (0.5h), especially the genes that were negatively affected by the heat stress. We next described the main steps a biologist took to identify such genes using XMAS (Figure 7).

The user chose to preserve the original 9 time points and used the median expression of all the involved samples to represent each time point. The bin size of 0.5 was used to discretize the data. Finally, the differentiation operator was used to subtract the control data from the stress data to facilitate cross-dataset comparison. The data analysis was started by interacting with a node corresponding to a negative differential at the 0.5h using the visualization of discretized trajectories An in-place window was activated, revealing that the node was associated with 29 probes (Figure 7(A)). Next, these 29 probes were isolated using the isolation filter (Figure 7(B)). Finally, the non-discretized trajectories of the 29 probes were obtained by calling the corresponding operator (Figure 7(C)). The trajectories were found to exhibit two principle patterns: one an increase in negative differential expression and the other pattern remaining static at the 0.5h expression level. Since the first pattern indicated that these genes were negatively affected by the heat stress, the full list of genes exhibiting this pattern was obtained by using the trajectory shape-based filter (Figure 7(D)). Following this step, detailed information was loaded for the visualized probes (Figure 7(E)). Information from PCAD was used at this point and it was established that many of the probes were replicates for the same gene. Furthermore, all the involved genes belonged to the ATP synthase functional group. This finding was found to agree with the results reported in [44].

### Case study 3: Analysis of structure-function relationships using PSPACE

We present three examples of protein annotation transfer and function inference using PSPACE. These examples demonstrate how PSPACE can be used to rapidly generate hypothesis about specific structure-function relationships, and reveal that particular structural insights which are dispersed across multiple data sources (e.g. Pfam, SCOP, PDB) may be recognized immediately through the experiential interface and interactions of PSPACE. These examples also illustrate how the experiential paradigm facilitates the use of expert intuition to guide search and analysis within the various protein data sources.

**Figure 8:**
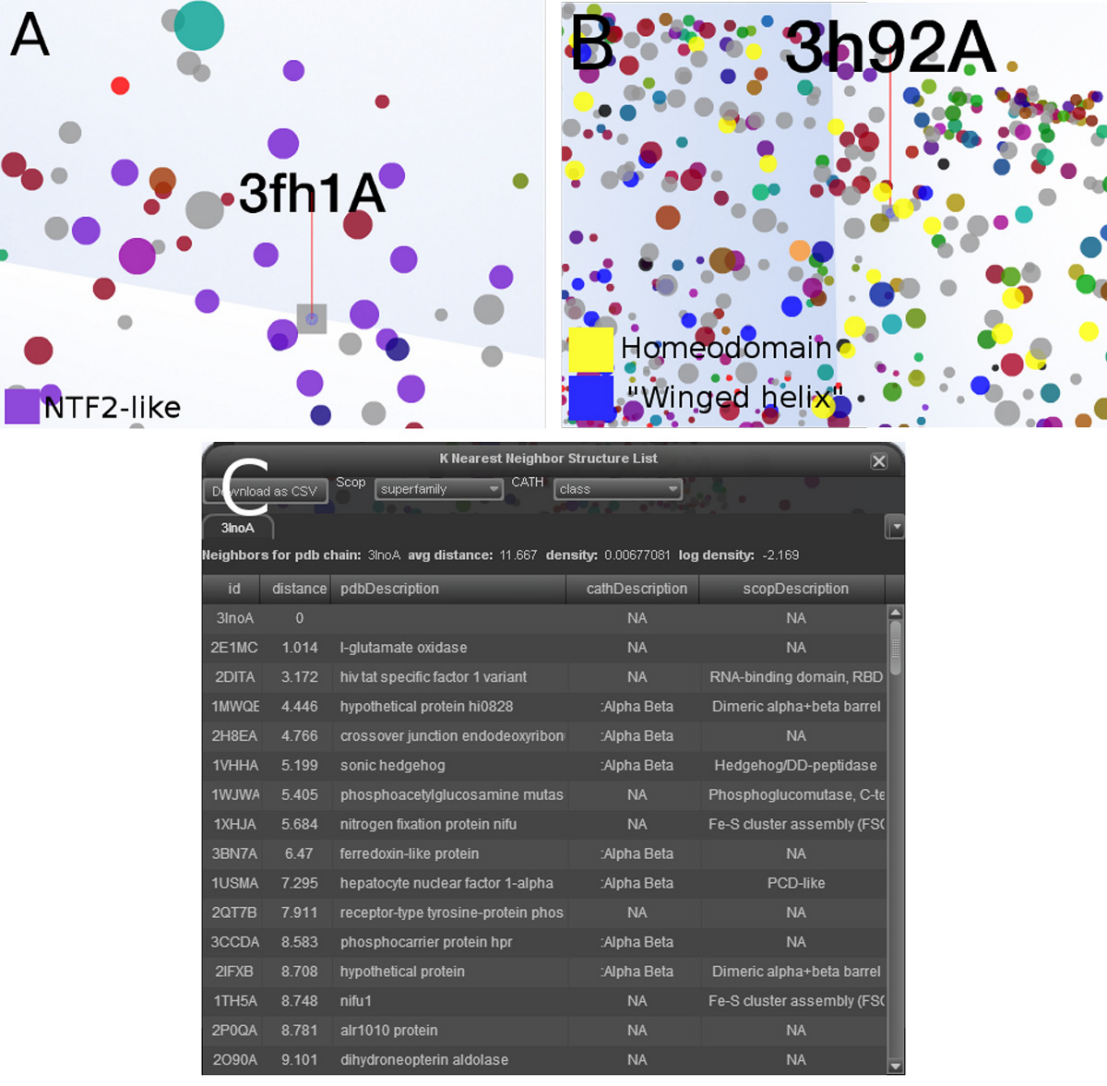
**Experiential function prediction using PSPACE**. Screenshots of PSPACE user interface elements during experiential function prediction. (A) Dense cluster of NTF-2-like chains surrounding 3FH1.A strongly suggests NTF-2-like designation. (B) Dispersed homeodomain and "winged helix" clusters around 3H92.A. (C) The k-nearest neighbors dialog populated with the structural neighborhood of 3LNO.A. Redox-active proteins in general and Fe-4S cluster assembly chains in particular are highly represented.

A relatively unambiguous example is given by the chain 3FH1.A, which has no known function, but occurs within a dense MPSS cluster corresponding to the NTF-2-like superfamily. While this information may be laboriously gleaned from the metadata of the molecule, the relation was immediately apparent within PSPACE (Figure 8A). The *k*-nearest neighbors dialog for this chain showed that the local region around 3FH1.A is enriched with many structures of the SnoaL-like polyketide cyclase family (not pictured). This assignment of 3FH1.A is supported by Pfam, which places 3FH1.A within a group of SnoaL-like polyketide cyclases implicated in nogalamycin biosynthesis (Pfam accession PF07366).

Another protein chain of unknown function, 3H92.A, is found in a band of protein space inhabited largely by two diverse superfamiles: the homeodomain-like and winged-helix DNA binding domain folds (Figure 8B). Most of its neighbors which are not members of these two groups are nevertheless members of other folds with the common function of nucleotide binding. Inspection of pairwise alignments between 3H92.A and its neighbors revealed that these structures align over a common core containing an apparent nucleotide binding motif. While PDB metadata for this chain reveals no more than that it may bind ATP, results from PSPACE were suggestive of a potential role for 3H92.A as a component of a transcriptional regulation complex.

The final example is 3LNO.A, a domain not listed by CATH or SCOP and classified as having an unknown function by both Pfam and GO. Its neighborhood in PSPACE (Figure 8C) contains many redox-active proteins as well as those involved in Fe-4S cluster assembly. Glutamate oxidase, in particular, is found very close to 3LNO.A, and several NifU C-terminal domain-like chains, as well as nucleotide binding structures, also occur in this vicinity. Inspections of individual alignments show that the core of 3LNO.A is highly similar to those from Fe-4S cluster assembly domains. Taken together, these results suggest that 3LNO.A may function as a module within a larger complex which engages in assembly of specific Fe-4S cluster-containing proteins.

### User studies

To examine the proposed design paradigm in terms of cognitive impact as well as in comparative settings, we conducted user studies of both applications. In these studies, XMAS was compared with two other open source non-experiential software systems for time series microarray data analysis, STEM [46] and Time Series Explorer [17] (TS-Explorer), while PSPACE was applied to two sets of protein structure analysis tasks and compared against either VisIT [47], a general visualization program, or the website of the RCSB PDB [48].

STEM consists of an interactive, sortable, visual representation of the output from its underlying clustering technique. It facilitates cross-datasets cluster comparison, and supports the specification of probe subsets that can be investigated within the context of the whole dataset. However, unlike XMAS, STEM does not support unified query-presentation spaces, direct operators, or a reflective interface. In TS-Explorer, coordinated visualizations (a primary scatter plot alongside secondary line charts) reflecting the activity of genes and changes in gene activity are used as an interface to explore the data. As in XMAS, users can directly interact with these visualizations through a unified and reflective interface. However, TS-Explorer supports limited analysis and is geared for finding unsuspected patterns of temporal activity.

The user study was designed to include both quantitative and qualitative evaluations. Specifically, we used the NASA Task Load Index (NASA-TLX) [49] to estimate workload complexity across the systems. NASA-TLX defines a mechanism to compute an overall workload score based on weighted scores for six workload factors. These six factors are: *mental demand*, *physical demand*, *temporal demand*, *effort*, *frustration*, and *performance*. The scores were mapped to an integer value between zero and ten, with zero denoting the best score. Our choice of TLX was prompted by the fact that the factors considered by TLX are directly related to the cognitive complexity of using complex systems like XMAS. Moreover, TLX has also been used by us to evaluate other experiential systems [4,5] and this experience helped us in appropriately designing and conducting the analysis.

#### XMAS

For XMAS, we designed seven information goal categories to mimic non-trivial pattern analysis tasks. These included: (1) finding temporally relevant features (e.g. identifying genes that increase or decrease their expression at specific time-points), (2) identifying genes that exhibit a shared expression pattern, (3) analysis of cross-data set features, (4) rapid validation of complex results, (5) exploratory analysis, (6) finding genes that exhibit periodicity in expression, and (7) identify genes that exhibit specific yet non-trivial expression patterns (e.g. genes showing stable expression in certain time periods, and increased expression at a subsequent time period).

**Figure 9:**
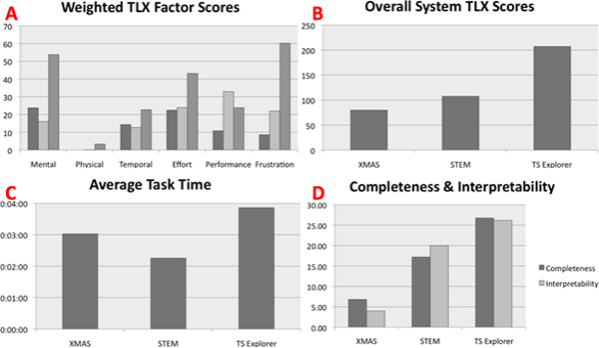
**Results from the user study involving XMAS, STEM and TS-Explorer**. (A) The weighted scores based on the six TLX factors, charted in the order of XMAS, STEM and TS-Explorer; (B) overall TLX scores; (C) average time taken to successfully conduct a task; and (D) completeness and interpretability of the three systems. Lower TLX scores indicate better performance.

The categories were designed to provide cross-system feature coverage and contained information analysis/exploration tasks of varying difficulty. Each category consisted of up to 6 instances of information goals. Ten users with graduate level education were recruited and each was assigned a set of five information goals using Latin squares to avoid assignment bias. A brief tutorial on all three systems was given to each participant before they attempted their tasks. Mouse clicks, task completion time and mouse pointer distance, were captured during evaluation to assess access complexity or ease of use. Each user was given a maximum of 4 minutes per task. After participants finished attempting the assigned tasks, they were asked to score the completeness and interpretability of the results. In Figure 9, we present the comparison results quantified in terms of the NASA-TLX factor scores, average time spent, and completeness and interpretability.

As can be seen from Figure 9, XMAS outperformed both STEM and TS-Explorer in terms of the overall TLX scores. The significance of the TLX scores based on ANOVA was *F *= 13.50 with *p *= 0.05. TS-Explorer was found to have performed poorly across all scoring factors with the exception of *performance*. Compared to XMAS, users found STEM less *mentally demanding*, but more *frustrating *and considered their *performance *worse within STEM as compared to XMAS. In terms of average task time also, STEM performed the best as tasks were generally completed with less interaction, less mouse movement and in less time. However, the limited functionality of STEM, led to poor user perception of the completeness and interpretability of results obtained through it. XMAS received relatively good scores across all factors, scoring most poorly in the *mental demand *and *effort *factors. User interviews conducted after the study indicated that due to the novelty of its data presentation and interaction paradigm, XMAS involved a steeper initial learning curve. This observation is consistent with our experience in evaluating other experiential systems [6]. However, in terms of completeness and interpretability, users rated XMAS significantly better than both STEM and TS-Explorer.

#### PSPACE

A user study in conjunction NASA-TLX was used to compare the subjective complexity of PSPACE with that of alternate software packages. In order to account for bias induced by increasing competency as experience is gained, tasks were presented to participants in permuted orders obtained from a balanced Latin square. All participants had academic backgrounds in biology.

The tasks used for evaluating PSPACE are summarized in Table 1. The first pair of tasks dealt with annotation and exploration of MPSS to characterize protein fold space in both global and local manners. For these tasks, users were asked to describe the general layout and spatial cohesion of either the primary protein classes (e.g. the top of SCOP), or two highly populated superfamilies (Immunoglobins and PH-domain superfamilies). As an alternate to PSPACE, study participants used VisIT, a desktop application which can be used for a large number of data-display operations (including interactive scatter plots) [47]. The subsequent tasks compared analysis modalities in terms of specific annotation tasks. The third task required participants to place two structures missing from either CATH or SCOP within their likely groups at each level of the un-annotated hierarchy, while the fourth had them assign potential functions to two proteins with no known functions as listed in PDB or other readily available sources. In addition to PSPACE, participants employed the web site of the PDB itself, which has been enhanced in recent years through integration with a large number of rich data sources, including structure and publication meta-data, annotation databases and pairwise structure alignments [48,50]. The tasks thus compared analysis modalities based essentially on either spatio-visual perception and exploration or textual analysis. Note that in practice such modalities are likely to be employed in a complimentary fashion rather than separately, as done in our study.

**Table 1:** Summary of tasks for TLX evaluation of PSPACE.

Task	Alternate	Time Allocated	Task Description
Charting Protein Classes	VisIT	10 min	Users inspect MPSS and need to determine the rough locations of protein classes at the top of CATH and SCOP hierarchies

Charting Protein Superfamiles	VisIT	10 min	As above, for two selected protein superfamiles: (Immunoglobins and PH-domain)

Structure Classification	RCSB PDB	15 min	Users are presented with two unclassified proteins and have to assign them likely classifications (PDB IDs 2cry.A, 2hvv.A)

Function Prediction	RCSB PDB	15 min	Users are presented with two proteins of unknown function and have to assign them likely functions (PDB IDs 3fh1.A, 3lno.A)

**Figure 10:**
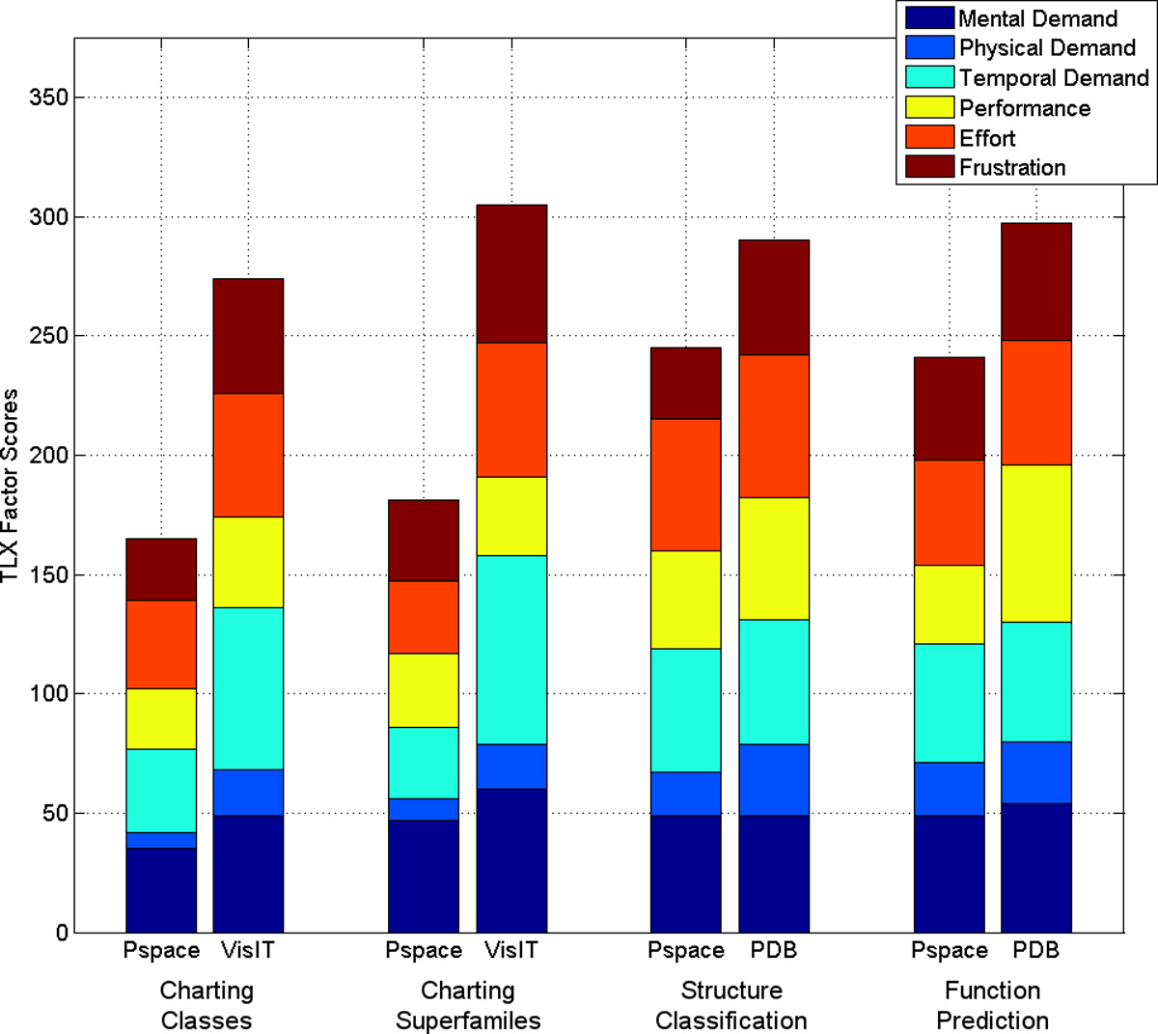
**TLX evaluation of PSPACE**. TLX Factor scores are shown as stacked bars. Each component's contribution to the total score is color coded as per the legend. Note that in TLX, lower scores (smaller bars) are defined to be better than higher scores, including for performance. Scores are population means, *n *= 7.

The population means (*n *= 7) of the TLX factor scores are presented in Figure 10. It is immediately apparent from the total height of the bars that PSPACE is generally superior in terms of user experience as measured by TLX. In particular, PSPACE was found to produce higher confidence results than alternatives, while requiring significantly less time and overall effort and inducing less frustration. The difference between PSPACE and VisIT is especially dramatic. Given that VisIT is a local application dedicated solely to visualization, these differences can be likely explained in terms of the domain-specific features included in PSPACE. For example, the large number of protein classification terms found at the lower levels of CATH and SCOP require that the color table for annotation of the MPSS be judiciously constructed to emphasize highly populated groups at the expense of the many singletons, lest important groups be assigned indistinguishable colors. PSPACE does this by relating the gap between discrete color levels to the population of a group; on the other hand a user of VisIT must make manual adjustments to the color codes for every classification term. Furthermore, loading and displaying MPSS with VisIT requires significantly more time and effort than in PSPACE.

The gap between TLX scores of PSPACE and PDB is smaller than that between corresponding scores of PSPACE and VisIT. This is because PSPACE and PDB provide complementary information. For the tasks dealing with "unknown annotations", PSPACE was considered consistently better than PDB in terms of *effort*, *frustration*, *performance* and *physical demand*. This observation is especially interesting given that most study participants already had significant experience using PDB. These data suggest that the experiential paradigm underlying PSPACE more effectively harnesses a user's cognitive capacity for pattern recognition than the iterative and primarily textual data presentation mode embodied by PDB. Furthermore, the experiential use of MPSS in PSPACE provides users with an immediate experience of inter-protein relationships at multiple scales, augmented by nearest neighbors search within a space capable of representing even "twilight-zone" structural similarity with high accuracy. This contrasts sharply with the "flat" representation found in PDB, where a user must read entries and inspect large numbers of high-scoring pairwise alignment partners in order to obtain a sense of a protein's structural neighborhood. Furthermore, pairwise alignment distributions of individual chains are often not adept at conveying distant relationships.

## Discussion

The research presented in this paper seeks to address critical design challenges for developing systems whose goal lies in facilitating analysis and sense-making with biological data. The design paradigm of experiential computing, which we espouse as a solution, seeks to combine ideas from information visualization, algorithmic data processing, and interactive data analysis. Our premise is that computers are inherently strong at large scale processing, data storage and data integration, but lack the human skills of contextual reasoning, hypotheses formulation, and sense making. Experiential computing seeks to combine the strengths of expert users with that of powerful visualization and algorithmic techniques.

Two concrete implementations (XMAS and PSPACE) of the experiential computing paradigm are described in this paper. In addition to providing practical software solutions for data analysis in their respective domains, these two systems highlight how the design principles of experiential computing can be translated to build real-world software. Using these systems as representatives, we have conducted quantitative evaluations of the proposed design paradigm and compared it to alternate strategies that currently exist. These evaluations were designed to assess both the efficacies of the participant systems (paradigms) as well as their usability. The results obtained by us demonstrate the proposed design paradigm to have important advantages. It is our hope that the design challenges, principles, and solutions described in this paper will facilitate the development of other systems that take advantage of human-computer synergy to address complex data exploration and sense-making tasks arising in life sciences.

## Competing interests

The authors declare that they have no competing interests.

## Authors' contributions

RS envisaged the application of experiential computing to biological data and designed the adaptation of the paradigm. BD, RS, HY, DA, and WM were involved in the development of XMAS. PSPACE was developed by DA, DF, and RS. BD, MG, SF, HY, and RS applied XMAS to the analysis of expression data at the human maternal interface. Analysis of heat stress on porcelain crabs with XMAS was done by BD, JS, HY, and RS. Experiments with PSPACE and its evaluation were done by DA, DF, and RS. The paper was written by RS, HY, and DA. Publication of this article was funded by the National Science Foundation grant IIS 0644418.

## Declarations

BD, DA, WM, and RS were funded in part by the National Science Foundation grant IIS 0644418 (CAREER). HY was funded in part by the CCLS mini grant program at San Francisco State University. JS was funded by the National Science Foundation grant BIO-ESS-OEI 0533920.

This article has been published as part of *BMC Bioinformatics *Volume 14 Supplement 14, 2013: Proceedings of the Tenth Annual MCBIOS Conference. Discovery in a sea of data. The full contents of the supplement are available online at http://www.biomedcentral.com/bmcbioinformatics/supplements/14/S14.
